# Changes in Epithelial and Stromal Corneal Stiffness Occur with Age and Obesity

**DOI:** 10.3390/bioengineering7010014

**Published:** 2020-02-07

**Authors:** Peiluo Xu, Anne Londregan, Celeste Rich, Vickery Trinkaus-Randall

**Affiliations:** 1 Department of Biomedical Engineering, Boston University, Boston, MA 02115, USA; angelax@bu.edu; 2 Department of Biochemistry/Molecular Biology, Boston University, Boston, MA 02215, USA; alondre@bu.edu; 3 Department of Biochemistry, Boston University School of Medicine, Boston, MA 02115, USA; cbrich@bu.edu; 4 Department of Ophthalmology, Boston University School of Medicine, Boston, MA 02115, USA

**Keywords:** basement membrane, confocal imaging, nanoindenter, pre-Type 2 diabetic

## Abstract

The cornea is avascular, which makes it an excellent model to study matrix protein expression and tissue stiffness. The corneal epithelium adheres to the basement zone and the underlying stroma is composed of keratocytes and an extensive matrix of collagen and proteoglycans. Our goal was to examine changes in corneas of 8- and 15-week mice and compare them to 15-week pre-Type 2 diabetic obese mouse. Nanoindentation was performed on corneal epithelium in situ and then the epithelium was abraded, and the procedure repeated on the basement membrane and stroma. Confocal imaging was performed to examine the localization of proteins. Stiffness was found to be age and obesity dependent. Young’s modulus was greater in the epithelium from 15-week mice compared to 8-week mice. At 15 weeks, the epithelium of the control was significantly greater than that of the obese mice. There was a difference in the localization of Crb3 and PKCζ in the apical epithelium and a lack of lamellipodial extensions in the obese mouse. In the pre-Type 2 diabetic obese mouse there was a difference in the stiffness slope and after injury localization of fibronectin was negligible. These indicate that age and environmental changes incurred by diet alter the integrity of the tissue with age rendering it stiffer. The corneas from the pre-Type 2 diabetic obese mice were significantly softer and this may be a result of changes both in proteins on the apical surface indicating a lack of integrity and a decrease in fibronectin.

## 1. Introduction

One area of great interest is understanding if the changes that occur to tissues and cells with disease or age reflect physical changes. The result of mechanical forces can be seen in endothelial vasculature [1], bone remodeling [2], and cell-cell communication between vascular endothelial cells on substrata [3]. In addition, when epithelial cells migrate they generally move as a sheet of cells to heal a wound rather than discrete cells and we hypothesize that the sheet generates unique forces on the underlying basement membrane depending on the stiffness of the substrate [4]. The motility of the sheet is enabled by protrusion of lamellipodia at the leading edge of the wound and requires a continual assembly and disassembly of focal adhesions that occur during extension, retraction, maturation of nascent adhesions [5].

The cornea provides a superb tissue to study changes in stiffness in epithelium, basement membrane proteins and stromal matrix proteins as it is accessible, and its properties can be readily examined. This tissue is constantly subjected to a wide range of mechanical stimuli. Response to the basement membrane is critical as changes in the composition of the basement membrane have been shown to mediate cell signaling associated with cell adhesion and migration [6,7]. Others have demonstrated that the degree of force generated by cells depends on substrate stiffness [8]. However, most of these studies were performed on single cells [9,10,11] or on monolayer cultures [12,13]. More recently Onochie et al., demonstrated that the leading-edge dynamics of migrating corneal epithelium were significantly different on a substrate of 8kPa compared to a stiffer substrate. In addition, there was a significantly greater phosphorylation of focal adhesion kinase proteins when cells were cultured and wounded on stiffer substrata [4].

The unwounded corneal epithelium consists of 5–7 layers of cells and the basal cells adhere to the basement membrane through structures called hemidesmosomes that are comprised of several large proteins that contact collagen anchoring fibrils in the anterior stroma. The stroma is comprised of an array of collagens and proteoglycans that permit the light to pass unaltered to the retina. Upon injury fibronectin is released and present transiently [14,15]. In addition, investigators have demonstrated that the matrix proteins change in the basement membrane zone of diabetic corneas with a decrease in laminin and an increase in heparan sulfate proteoglycans [16].

Here we investigate if the change in stiffness of corneal epithelium, basement membrane zone and stroma are altered either with age or in an obese mouse that is a model for a Type 2 pre-diabetes (DiO; diet induced obesity). Previously, investigators demonstrated that the DiO mice had impaired skin and corneal wound healing and a change in the regulation of an ionotropic receptor, P2X7 [17,18].

In our current study we found a significant increase in epithelial stiffness with age and an age matched decrease in the DiO epithelium. In contrast, there is a greater percent change in stiffness in the corneal stroma (anterior to posterior) in the 8-week mice compared to the 15-week mice. Furthermore, there is little if any change in stiffness in the stroma in the 15-week DiO mice. To understand these changes, we examined the polarity protein, Crumbs3, that is associated with tight junctions in the epithelium. Together our results demonstrate that age and environment impact the integrity of the cornea.

## 2. Materials and Methods

### 2.1. Chemicals

Anti-fibronectin monoclonal mouse antibodies (clone FN-3E2, Lot# 104M4800V) were purchased from Sigma-Aldrich (St. Louis, MO, USA). Rhodamine phalloidin (Lot# 1842723) was purchased from Invitrogen by Thermo Fisher Scientific (Waltham, MA, USA). Alexa Fluor 488 goat anti-mouse was obtained from Invitrogen by Thermo Fisher Scientific (Waltham, MA, USA). VectaSHIELD with 4′,6-diamidino-2-phenylindole (DAPI) was purchased from Vector Labs (Burlingame, CA, USA).

### 2.2. Tissue Preparation

The research protocol conformed to the standards of the Association for Research in Vision and Ophthalmology for the Use of Animals in Ophthalmic Care and Vision Research and the Boston University Institutional Animal Care and Use Committee (IACUC). Control and DiO mice were obtained from Jackson Laboratory (The Jackson Laboratory; Bar Harbor, ME, USA). Control mice were maintained on the diet, D1425OB (10 kcal% fat, 3.8 kcal/g), while the DiO mice were fed a high fat diet, D12492 for 15 weeks (60 kcal% fat, 5.2 kcal/g). The mice exhibited obesity (40.8 g versus 32.4 g at 16 weeks), an elevated HbA1c, mild hyperglycemia, significantly elevated triglycerides, impaired glucose tolerance, and blood glucose for DiO that remained elevated at 120 min. In addition, no difference in bone mineral density in DiO mice was detected. Prior to delivery, body weight, blood glucose and other measurements were performed as described [1,2,3,4,5,6,7,8,9].

All eyes were examined under a microscope prior to experiments to make sure there were no ocular injuries prior to debridement. Experiments were performed ex vivo and all animals were sacrificed prior to measurements or injury. To examine localization of proteins, a debridement wound was made with a dull laser blade following demarcation with a 1.5 mm-diameter trephine in the central cornea [19]. After wounding, the eyes were incubated in Dulbecco’s Modified Eagles medium (DMEM) at 37 °C and 5% CO_2_ for different time points. After incubation, the eyes were fixed with 4% paraformaldehyde for 30 min at room temperature. The tissue was then dissected, leaving an intact scleral rim, and cut into radial wedges.

### 2.3. Nanoindentation

Corneal epithelium and stromal stiffnesses were measured with a Piumananoindenter system (Optics11, Amsterdam, the Netherlands). To perform the measurements the head was immobilized. The eye was immersed in phosphate buffered saline (PBS) and the probe was lowered onto the center of the cornea. The probe had a tip radius of 26 μm and stiffness of 4.4 N/m. Epithelium measurements were taken through 10 μm indentations.

To examine the basement membrane and stroma, the epithelium was abraded. Measurements were taken using various indentation depths, ranging from 1 μm to 17 μm. The Young’s modulus of the sample was calculated using the built-in PIUMA software based on the Hertzian contact mechanics model, assuming cornea tissue is perfectly incompressible with a Poisson ratio of 0.5. The loading curves were fit to the following equation by the software:

$$F=\;\frac{4}{3}\;E_{eff}\;\sqrt{R_{i}}\;\cdot h^{\frac{3}{2}}$$

where F represents the applied force, $E_{eff}$ represents the effective Young’s modulus, $R_{i}$ represents the spherical tip radius, and h represents indentation depth. The bulk Young’s modulus we used for analysis was generated using the following equation:

$$E=\;E_{eff}\;(1-\nu^{2})$$

where ν represents Poisson’s ratio of the measured material.

### 2.4. Immunohistochemistry

Tissues were permeabilized with 0.1% *v*/*v* Triton X-100 in PBS and blocked with 4% bovine serum albumin (BSA) solution in PBS for 1 h for indirect immunofluorescence. Samples were incubated in anti-fibronectin solution (1:200, Sigma-Aldrich, St. Louis, MO, USA), or anti-laminin-5 (γ2 chain) (1:200, MilliporeSigma, Burlington, MA, USA) at 4 °C overnight, washed with PBS, and incubated in Alexa-Fluor-conjugated secondary antibodies (1:200; Invitrogen by Thermo Fisher Scientific, Waltham, MA, USA) for 1 hr. Samples were washed with PBS, then incubated in rhodamine phalloidin (1:50, Invitrogen by Thermo Fisher Scientific, Waltham, MA, USA) for 20 min.

### 2.5. Confocal Microscopy

Tissues were mounted using VectaSHIELD antifade mounting medium with DAPI during image acquisition. Tissues were placed apical side down on a glass coverslip bottom petri dish and flattened by placing another glass coverslip on top of the tissue. Tissues were imaged using a 40× oil immersion objective. The gain and laser intensity were set according to a secondary control sample and remained constant throughout the experiment. The pinhole was maintained at 1 airy unit for all sample images. The slice interval for all z-stack images was 1 μm.

### 2.6. Statistical Analysis

Values were presented as the mean ± standard error of the mean (SEM). Statistical significance was determined by the Wilcoxon rank-sum test (also known as the Mann-Whitney U Test) using the MATLAB function.

## 3. Results

The corneal epithelium is typically a very stable structure due to tight junctions that are located in the apical epithelium that prevent growth factors and other molecules from penetrating, binding to their receptors, and activating signaling cascades [14].

### 3.1. Stiffness Is Age and Obesity Dependent

**Figure 1:**
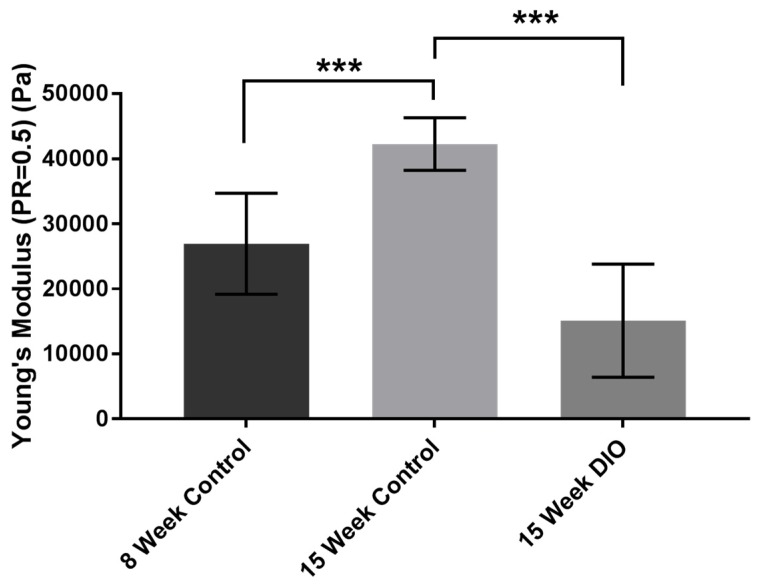
Corneal epithelium stiffness (mean ± standard deviation) for 8-week control, assuming cornea tissue is perfectly incompressible with a Poisson ratio of 0.5.

In the following experiments we compared mice of 8 and 15 weeks with a naturally occurring murine obesity model (pre-Type 2 diabetic mice) to examine changes in stiffness in epithelium and the stroma. Both eyes from five 8-week control mice, five 15-week control mice, and 2 DiO 15-week-old mice were used. Previously, we demonstrated that corneal epithelial wound repair is impaired in corneas from the obese mice [19]. These results stimulated us to examine changes in the cornea that might be underlying causes of the changes in the cell migration and wound repair. In Figure 1 we examined the stiffness of epithelium, basement membrane, and stroma in intact eyes. Young’s modulus was calculated and the intact corneal epithelium from a 15-week C57Bl6 mouse was significantly (Wilcoxon rank-sum test, *** *p* < 0.01) greater than the epithelium from an 8-week mouse. At the equivalent age, the epithelium was significantly (Wilcoxon rank-sum test, *** *p* < 0. 01) greater than an age matched DiO mouse cornea. Here the background of the mice are similar and the DiO mouse is fed a high fat diet for 15 weeks.

### 3.2. Localization of Polarity Proteins

**Figure 2:**
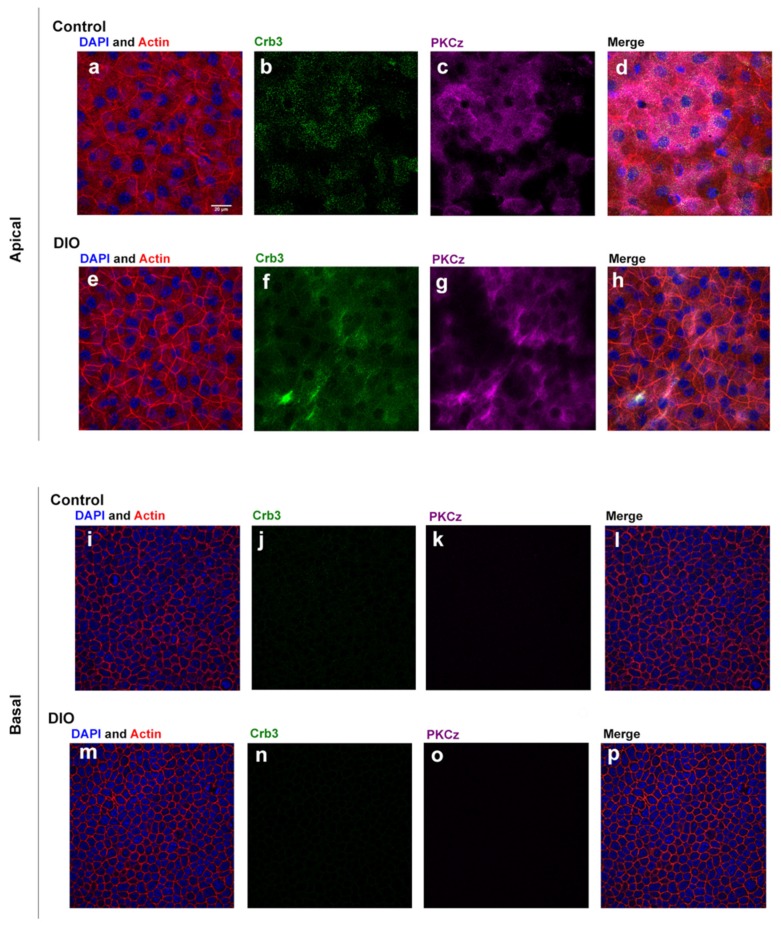
Localization of Crb3 and PKCζ in unwounded control and DiO corneal epithelium. An apical and a basal image are displayed for each condition. Tissue was stained for Crb3 and PKCζ and counter stained with rhodamine phalloidin and DAPI. Apical cells at z-plane 5 out of 25 slices, imaged at 1μm interval, are shown (**i**–**p**). Both the control (**j**,**k**) and DiO (**n**,**o**) tissue show no staining for either Crb3 or PKCζ (Control apical-(**a**). DAPI and actin, (**b**). Crb3, (**c**). PKCζ, (**d**) merged; DiO apical-(**e**). DAPI and actin, (**f**). Crb3, (**g**). PKCζ, (**h**) merged; Control basal-(**i**). DAPI and actin, (**j**). Crb3, (**k**). PKCζ, (**l**). merged; DiO basal-(**m**). DAPI and actin, (**n**). Crb3, (**o**). PKCζ, (**p**) merged).

Since there was a major difference in stiffness in the control and DiO epithelium, we examined localization of a polarity protein, Crumbs3 (Crb3), which is associated with epithelial tight junctions and recruits tight junction proteins, such as ZO-1, to tight junction structures. Crumbs3 has been shown to maintain apical-basal polarity, apical stability, cell adhesion, and epithelial integrity [20] and is required for airway differentiation. We hypothesized that we would find changes in Crb3. Images are presented in 2 ways (Figure 2).

**Figure 3:**
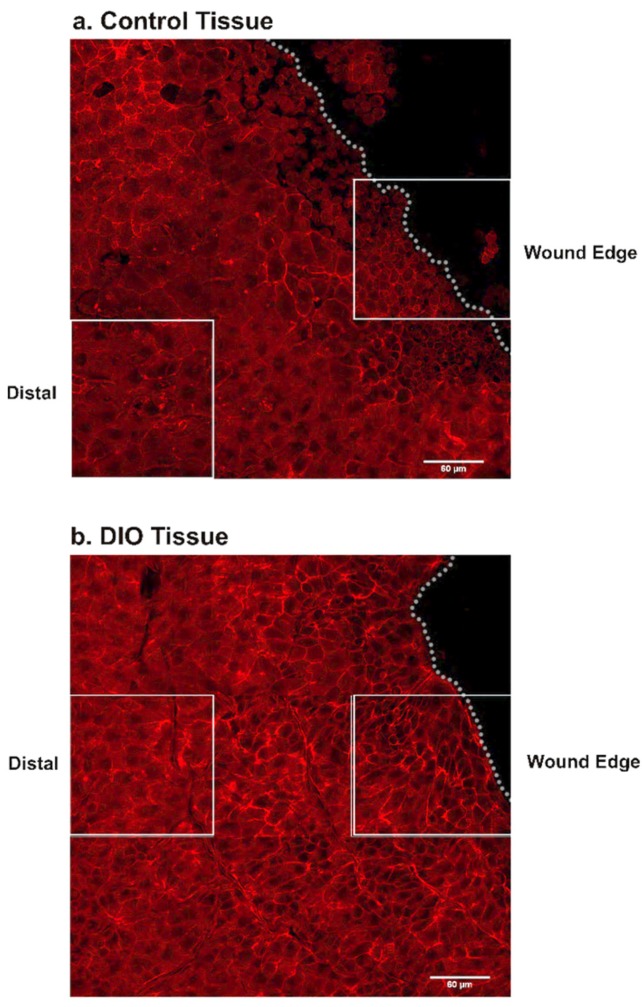
Wound healing is reduced in DiO tissue stained for Crb3 and PKCζ. A tiled image was taken comprised of 20 slices taken at 0.5 μm intervals; shown is z-plane 4 stained only for actin. The z-stack used as a representative image at the wound edge and distal to the wound edge is indicated by the white box. Tissue shown was also stained for Crb3 and PKCζ. The wound edge is indicated by the white dotted line. (**a**) In control tissue apical cells can be identified distal to the wound by their larger polygonal shape while the cells along the wound edge are smaller and more uniform, indicating that they are basal cells which have collectively migrated over the wound area. (**b**) In DiO tissue, apical cells are also apparent distal to the wound, but along the wound edge there is reduced basal cell migration.

**Figure 4:**
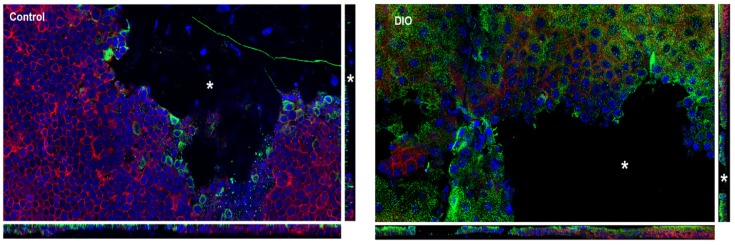
Change in Crb3 after epithelial injury. Maximal projection images were taken, and orthogonal images made. The side bars show cross-sections with an apical-basal view of Crb3 (green), DAPI (blue) and actin (red). Asterisk denotes wound.

Tissue was stained for Crb3 and PKCζ and counter stained with rhodamine phalloidin and DAPI and an image is shown of the most apical image and of a basal image. Both Crb3 and PKCζ are present in apical images but localization is different. The control tissue shows punctate staining of Crb3 (Figure 2b) and PKCζ (Figure 2c). The DiO tissue shows diffuse staining for both Crb3 (Figure 2f) and PKCζ (Figure 2g). In addition, there is no staining of either in the basal layer. To examine changes in localization after injury we first examined F-actin at the edge of the wound (Figure 3) and demonstrated that the morphology of the cells is different. We then examined the localization of Crb3 and performed maximal projection images and made orthogonal sections to examine apical polarity in cross section (Figure 4).

**Figure 5:**
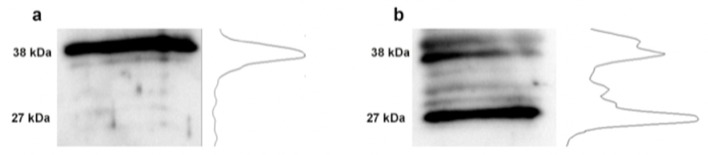
Glycosylation of Crb3 in control and DiO corneal epithelium. Protein extracts treated with PNGase and then were run on a 12% SDS-PAGE gel. The protein was transferred overnight to a nitrocellulose membrane for Western blotting. The blot was then probed for Crb3. The associated line graphs are indicative of the relative densities within the lane. (**a**) Control; (**b**) DiO.

When the control tissue was treated with PNGase we found the major form of Crb3 at 38kDa. Likewise, when the DiO tissue was treated with PNGase the major form of Crb3 shifted to 27kDa, indicating that Crb3 is more highly glycosylated. The major 38kDa form is also present (Figure 5).

### 3.3. Stiffness of Corneal Basement Membrane and Stroma

**Figure 6:**
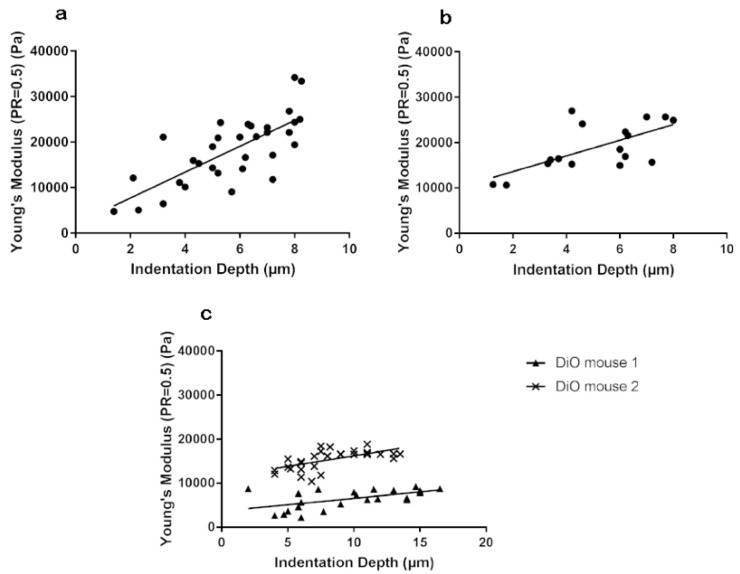
Corneal stromal stiffness. Young’s modulus (Pa) was determined throughout the depth of the stroma in control 8- and 15-week corneas and compared to 15-week DiO corneas. (**a**) 8-week control (R^2^ = 0.5527) (**b**) 15-week control (R^2^ = 0.4534) (**c**) 15-week DiO (R^2^ = 0.311 for DiO mouse 1; R^2^ = 0.3744 for DiO mouse 2). Both eyes were measured for each mouse.

We abraded the corneas, removed the epithelium, and indented at different depths to examine the differences in corneal basement membrane and stromal stiffness. We have shown previously using electron microscopy that the basement membrane is intact [21]. Data was collected from five control 8-week mice, five control 15-week mice, and two DiO mice. Results have shown that basement membrane and stromal stiffness shows an increasing trend as indentation depth increases in control mice and is similar among different individuals (Figure 6). However, this trend is not as obvious in DiO mice and has greater variance between individuals.

### 3.4. Changes in Fibronectin

**Figure 7:**
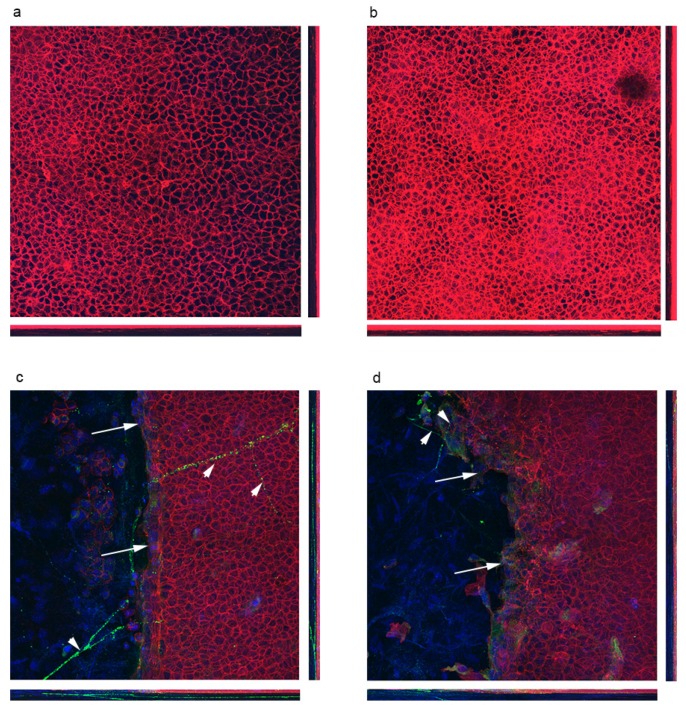
Corneas stained for fibronectin and counter stained with rhodamine phalloidin. 40 μm of 2 × 2 tile z-sections were taken and presented as maximum intensity with orthogonal sections. (**a**) 15-week unwounded control (**b**) 15-week unwounded DiO (**c**) 15-week control 20 h after wounding (**d**) 15-week DiO 20 h after wounding.

We examined for changes in fibronectin as it is known to be transiently localized in the stroma and wound edge of control corneas. In the control cornea we found that fibronectin was present at the wound edge (arrows). In addition, we detected fibronectin along the stromal nerves in the control corneas (arrowheads) where it is presumably secreted by the nerves (Figure 7a). The stromal branch is detected in the orthogonal image. In contrast, in the DiO cornea fibronectin is diffuse (arrow) and does not appear in an organized manner along the wound edge (Figure 7b). In addition, the staining along the stromal nerve is negligible (arrowheads). As the stromal nerves are present in the 15-week DiO mouse cornea [19], we hypothesized that the secretion is defective. Fibronectin was not detected in the control unwounded cornea (Figure 7c) as has been described previously [15,22].

## 4. Discussion

The cornea is unique because it serves an essential function as the strongest refracting surface of the eye while also maintaining an impermeable barrier between the eye and the external environment. We have shown that changes in stiffness occur with age as well as on obese mice (DiO) that are a model of pre-Type II diabetes. Previously, we demonstrated that corneas of DiO mice heal significantly slower than corneas from control C57BL6 mice [19]. Our data in this manuscript support a report that pre-diabetics have an increased incidence of corneal surface disorders compared to controls (20.67% vs. 3.33%, respectively; *p* < 0.05) [23].

We demonstrated that the stiffness of epithelium was age dependent and diet dependent for age matched animals. As the epithelium of the DiO animals was not as stiff we reflected on the changes in integrity of the epithelium and stained for polarity proteins that are typically seen in the apical epithelium. We examined unwounded and wounded corneas as planar polarity can be initiated by several different cues, including changes in growth factors and/or the extracellular matrix [16]. The cues are thought to be converted into directional migration, which requires the reorganization of the cellular components by signaling pathways. These proteins generally become localized to the front of the migrating cells resulting in cytoskeletal changes with membrane protrusions at the leading edge and directional movement [24]. These changes were observed in the control and were modified in the DiO corneas. Previously, in other tissue Crb3 was found to localize to the apical surface in both differentiated secretory and differentiated ciliated cells [20].

Differences in stiffness were also detected in the basement membrane and stroma with a change in the percent change of stiffness through the stroma. All the measurements were made in the central cornea. The reason for the change in stiffness is not well understood [25]. showed that with age connective tissue is characterized by an accumulation of Advanced Glycation End-Products (AGEs) and that states that diabetics are impacted more by their accumulation. This is important as it can alter the glycation of proteins such as collagen that are abundant in the stroma. Their study on tendons in vitro demonstrated that AGEs reduce the viscoelasticity of the tissue. The data on the accumulation of AGEs over time and their impact on collagen fibrils and the spacing of proteoglycans is not known.

In a recent review, McKay et al., stated that collagen cross-linking provides the strength for maintaining the integrity of the cornea and that aging and diabetes are associated with an increase in collagen cross-linking [26]. However, the review then stated that while corneal hysteresis and corneal resistance factor were elevated in the diabetic population there were inconsistencies and they proposed it was due to the heterogeneity of the human patient population. Consistency within a diabetic population is extremely difficult to control for several reasons including severity and length of the disease. For this reason, study of several different mouse diabetic models over time will be interesting to resolve this question. McKay did report increased stromal thickness with none to moderate increase in stiffness depending on the studies. In our studies we found that there was an increase in stromal thickness in the DiO cornea Kneer et al. (2018).

Other investigators hypothesized that the increase was associated with diabetes, and found instead that there was no association of lysyl oxidase (LOX) with diabetes or obesity in their cohort [27]. While we measured stiffness, we did not measure LOX and its expression needs to be examined in the DiO mouse at different times of diet. In fact, their in vitro studies showed a greater association of elevated LOX with hypoxia. Furthermore, Mankus et al. (2012) demonstrated in the P2X7 knock out mouse that LOX was elevated as was Type III collagen and was not indicative of increased cross-linking [28]. However, in the DiO mouse and in diabetic human corneas, P2X7 mRNA is significantly elevated 7–10 fold [19,28]. The functional meaning of this elevated expression is not yet understood. In our current studies collagen fibril measurements were not performed, and future studies will further examine the extracellular matrix of the stroma. It is possible that there is a change in the sulfation of proteoglycans as this could also affect the stiffness.

Our studies propose that the impaired wound healing in the DiO cornea could reflect the difference in stiffness or a change in the matrix molecules such as fibronectin. In the control the fibronectin was detected along the stromal nerves and at the leading edge of control compared to the DiO corneas. A reduction in fibronectin was detected in corneas subjected to hypoxia, where healing was delayed compared to control [15,22,29]. It is even possible that the lack of compliancy reflects the decrease in fibronectin as in vitro studies have shown that stretching of fibronectin causes it to unfold leading to exposure of cryptic binding sites [30]. The usage of the nanoindenter on mice of the same background at different ages and environmental stresses provides a controlled study for a field that has much conflicting data because the human model is so complex. Additional studies over time and on Type 2 diabetic models of obese and non-obese mice will be conducted to further test our results. Identification of specific factors that alter the integrity of the cornea will be used in treating and preventing changes. In summary, study of the physical features of the cornea such as stiffness in situ provides insight into the mechanisms of wound repair in control and diseased conditions.

## References

[1] Resnick N., Yahav H., Shay-Salit A., Shushy M., Schubert S., Zilberman L.C., Wofovitz E. (2003). Fluid shear stress and the vascular endothelium: For better and for worse. Prog. Biophys. Mol. Biol..

[2] Orr A.W., Helmke B.P., Blackman B.R., Schwartz M.A. (2006). Mechanisms of mechanotransduction. Dev. Cell.

[3] Derricks K.E., Trinkaus-Randall V., Nugent M.A. (2015). Extracellular matrix stiffness modulates VEGF calcium signaling in endothelial cells: Individual cell and population analysis. Integr. Biol..

[4] Onochie O.E., Onyejose A.J., Rich C.B., Trinkaus-Randall V. (2019). The Role of Hypoxia in Corneal Extracellular Matrix Deposition and Cell Motility. Anat. Rec..

[5] Petrie R.J., Yamada K.M. (2012). At the leading edge of three-dimensional cell migration. J. Cell Sci..

[6] Sazonova O.V., Lee K.L., Isenberg B.C., Rich C.B., Nugent M.A., Wong J.Y. (2011). Cell-cell interactions mediate the response of vascular smooth muscle cells to substrate stiffness. Biophys. J..

[7] Mammoto T., Ingber D.E. (2010). Mechanical control of tissue and organ development. Development.

[8] Roca-Cusachs P., Iskratsch T., Sheetz M.P. (2012). Finding the weakest link—Exploring integrin-mefiated mechanical molecular pathways. J. Cell Sci..

[9] Chen Y.C., Allen S.G., Ingram P.N., Buckanovich R., Merajver S.D., Yoon E. (2015). Single-cell migration chip for chemotaxis-based microfluidic selection of heterogeneous cell populations. Sci. Rep..

[10] Gardel M.L., Schneider I.C., Aratyn-Schaus Y., Waterman C.M. (2010). Mechanical Integration of Actin and Adhesion Dynamics in Cell Migration. Annu. Rev. Cell Dev. Biol..

[11] Mak M., Spill F., Kamm R.D., Zaman M.H. (2016). Single-cell migration in complex microenvironments: Mechanics and signaling dynamics. J. Biomech. Eng..

[12] Tambe D.T., Hardin C.C., Angelini T.E., Rajendran K., Park C.Y., Serra-Picamal X., Zhou E.H., Zaman M.H., Butler J.P., Weitz D.A. (2011). Collective cell guidance by cooperative intercellular forces. Nat. Mater..

[13] Brugues A., Anon E., Conte V., Veldhuis J.H., Gupta M., Colombelli J., Munoz J.J., Brodland G.W., Ladoux B., Trepat X. (2014). Forces driving epithelial wound healing. Nat. Phys..

[14] Lee A., Karamichos D., Onochie O.E., Hutcheon A.E.K., Rich C.B., Zieske J.D., Trinkaus-Randall V. (2018). Hypoxia modulates the development of a corneal stromal matrix model. Exp. Eye Res..

[15] Blanco-Mezquita J.T., Hutcheon A.E., Stepp M.A., Zieske J.D. (2011). alphaVbeta6 integrin promotes corneal wound healing. Investig. Ophthalmol. Vis. Sci..

[16] Ljubimov A.V. (2017). Diabetic complications in the cornea. Vis. Res..

[17] Minns M.S., Teicher G., Rich C.B., Trinkaus-Randall V. (2016). Purinoreceptor P2X7 Regulation of Ca(2+) Mobilization and Cytoskeletal Rearrangement Is Required for Corneal Reepithelialization after Injury. Am. J. Pathol..

[18] Dimitriadis E.K., Horkay F., Maresca J., Kachar B., Chadwick R.S. (2002). Determination of elastic moduli of thin layers of soft material using the atomic force microscope. Biophys. J..

[19] Kneer K., Green M.B., Meyer J., Rich C.B., Minns M.S., Trinkaus-Randall V. (2018). High fat diet induces pre-type 2 diabetes with regional changes in corneal sensory nerves and altered P2X7 expression and localization. Exp. Eye Res..

[20] Szymaniak A.D., Mahoney J.E., Cardoso W.V., Varelas X. (2015). Crumbs3-mediated polarity directs airway epithelial cell fate through the hippo pathway effector Yap. Dev. Cell.

[21] Payne J., Gong H., Trinkaus-Randall V. (2000). Tyrosine phosphorylation: A critical component in the formation of hemidesmosomes. Cell Tissue Res..

[22] Lee A., Derricks K., Minns M., Ji S., Chi C., Nugent M.A., Trinkaus-Randall V. (2014). Hypoxia-induced changes in Ca(2+) mobilization and protein phosphorylation implicated in impaired wound healing. Am. J. Physiol. Cell Physiol..

[23] Zaleska-Żmijewska A., Piątkiewicz P., Śmigielska B., Sokołowska-Oracz A., Wawrzyniak Z.M., Romaniuk D., Szaflik J., Szaflik J.P. (2017). Retinal photoreceptors and microvascular changes in prediabetes measured with adaptive optics (rtx1^TM^): A case-control study. J. Diabetes Res..

[24] Collins C., Nelson W.J. (2015). Running with neighbors: Coordinating cell migration and cell-cell adhesion. Curr. Opin. Cell Biol..

[25] Gautieri A., Passini F.S., Silván U., Guizar-Sicairos M., Carimati G., Volpi P., Moretti M., Schoenhuber H., Redaulli A., Berli M. (2017). Advanced glycation end-products: Mechanics of aged collagen from molecule to tissue. Matrix Biol..

[26] McKay T.B., Priyadarsini S., Karamichos D. (2019). Mechanisms of collagen crosslinking in diabetes and keratoconus. Cells.

[27] Pastel E., Price E., Sjöholm K., McCulloch L.J., Rittig N., Liversedge N., Knight B., Møller N., Svensson P.A., Kos K. (2018). Lysyl oxidase and adipose tissue dysfunction. Metabolism.

[28] Mankus C., Chi C., Rich C., Ren R., Trinkaus-Randall V. (2012). The P2X_7_ receptor regulates proteoglycan expression in the corneal stroma. Mol. Vis..

[29] Onochie O.E., Zollinger A., Rich C.B., Smith M., Trinkaus-Randall V. (2019). Epithelial cells exert differential traction stress in response to substrate stiffness. Exp. Eye Res..

[30] Foolen J., Shiu J.Y., Mitsi M., Zhang Y., Chen C.S., Vogel V. (2016). Full-length fibronectin drives fibroblast accumulation at the surface of collagen microtissues during cell-induced tissue morphogenesis. PLoS ONE.

